# Immune sentinel function of nasal mucosal epithelial cells in allergic rhinitis: a review on barrier damage and inflammatory amplification loops regulated by calcium signalling

**DOI:** 10.3389/fimmu.2026.1761813

**Published:** 2026-03-26

**Authors:** Yiting Liu, Qingjia Sun, Cuida Meng, Jichao Sha, Dongdong Zhu, Nan Wu

**Affiliations:** 1 Department of Otolaryngology Head and Neck Surgery, China-Japan Union Hospital of Jilin University, Changchun, China; 2 Jilin Provincial Key Laboratory of Precise Diagnosis and Treatment of Upper Airway Allergic Diseases, Changchun, China; 3 Otolaryngology Head and Neck Surgery Research Center, Changchun, China; 4 Phase I Clinical Trial Research Laboratory, China-Japan Union Hospital of Jilin University, Changchun, China

**Keywords:** allergic rhinitis, barrier damage, calcium signaling, immune sentinel, inflammatory amplification loop, nasal mucosal epithelial cells

## Abstract

Allergic rhinitis (AR) is a prevalent chronic inflammatory disorder characterized by complex pathophysiological mechanisms. Nasal mucosal epithelial cells serve as crucial “immune sentinels” that detect allergens and initiate immune responses, thereby playing a pivotal role in disease progression. Intracellular calcium signaling, as a vital second messenger, regulates epithelial barrier integrity and modulates immune functions within these cells. This review summarizes current understanding of the immune surveillance role of nasal mucosal epithelial cells in AR, emphasizing the regulatory mechanisms of calcium signaling pathways in barrier disruption and the amplification of inflammatory cycles. Recent studies reveal that aberrant calcium signaling contributes to nasal epithelial barrier dysfunction and excessive activation of inflammatory cells, which perpetuate chronic inflammation and exacerbate symptom severity. By integrating emerging evidence on calcium-mediated cellular processes, this article highlights the critical involvement of calcium signaling in maintaining epithelial homeostasis and controlling inflammatory responses in AR. Understanding these mechanisms provides novel insights into the pathogenesis of AR and identifies potential therapeutic targets aimed at restoring epithelial barrier function and modulating inflammatory cascades, thereby offering new directions for clinical intervention.

## 1 Introduction

Allergic rhinitis (AR), characterized by nasal congestion, sneezing, itching, and rhinorrhea, imposes substantial socioeconomic burdens worldwide (1, 2). The nasal epithelium functions as the first line of defense, sensing and responding to external stimuli, including allergens, pollutants, and pathogens, thereby orchestrating local immune responses to maintain mucosal homeostasis (3).Recent advances have expanded the conceptualization of the nasal epithelium beyond a mere physical barrier to that of an “immune sentinel” or “immune watchman.” (4) Nasal mucosal epithelial cells detect noxious stimuli through pattern recognition receptors (PRRs) and other sensor molecules, subsequently releasing cytokines, chemokines, and alarmins that modulate the activity of innate and adaptive immune cells such as group 2 innate lymphoid cells (ILC2s), eosinophils, and T lymphocytes (5). However, in AR, this finely tuned balance is disrupted, leading to epithelial barrier impairment and exaggerated inflammatory responses (6, 7). Central to the regulation of epithelial cell function and immune responses is intracellular calcium (Ca^2+^) signaling, which serves as a ubiquitous second messenger controlling diverse cellular processes including barrier maintenance, cytokine secretion, and cell-cell communication (8, 9). Aberrant Ca^2+^ signaling has been increasingly recognized as a key contributor to epithelial barrier dysfunction and the amplification of inflammatory cascades in allergic diseases (10). This review aims to systematically synthesize current knowledge on the immune surveillance functions of nasal epithelial cells in AR, with a particular focus on the regulatory role of Ca^2+^ signaling in barrier disruption and inflammatory amplification. By elucidating these pathways, we hope to contribute to a deeper mechanistic insight into AR pathophysiology and foster the development of targeted therapeutic strategies that restore epithelial barrier function and modulate aberrant immune responses, ultimately improving clinical outcomes for patients suffering from this pervasive allergic condition (6, 11, 12). Building on these well-characterized research gaps, the present review is the first to put forward a novel conceptual model—the Ca²^+^-mediated epithelial gatekeeper axis—for AR. At its core, this model holds that nasal mucosal epithelial cells, the key mucosal immune sentinels in the upper airway, initiate Ca²^+^ signaling cascades upon allergen recognition via PRRs, and in turn drive AR pathogenesis through a dual regulatory mechanism: direct modulation of epithelial tight junction integrity and activation of the nuclear factor kappa-light-chain-enhancer of activated B cells (NF-κB)/NLR family pyrin domain containing 3 (NLRP3) inflammatory signaling pathways. This signaling cascade ultimately gives rise to a self-sustaining vicious cycle in AR pathogenesis: aberrant Ca²^+^ signaling → epithelial barrier impairment → inflammatory amplification → exacerbated Ca²^+^ signaling dysregulation. This integrated mechanistic framework thus serves as a central tenet for elucidating the chronic pathological process of AR.

## 2 Physiological functions of nasal mucosal epithelial cells as “immune sentinels”

The nasal mucosal epithelium constitutes a complex, multilayered cellular architecture that serves as the primary physical barrier against inhaled environmental insults, including allergens, pathogens, and particulate matter (13). These tight junctions (TJs) form a dynamic seal that is responsive to environmental and immunological stimuli, ensuring selective permeability while preserving mucosal homeostasis (14). Disruption of this barrier function is increasingly recognized as a pivotal event in the pathogenesis of AR and other sinonasal inflammatory diseases (15).

Nasal mucosal epithelial cells serve as critical immune sentinels by detecting allergens and pathogens through an array of PRRs, which are essential for initiating immune responses in AR (16). These PRRs, including Toll-like receptors (TLRs) and nucleotide-binding oligomerization domain (NOD)-like receptors, recognize conserved molecular patterns on pathogens (PAMPs) or damage signals (DAMPs), thereby triggering intracellular signaling cascades that activate innate immunity (17). These alarmins are pivotal in initiating type 2 inflammation characteristic of allergic diseases by activating ILC2s and promoting Th2 responses (18, 19). This epithelial-driven mechanism of type 2 inflammatory initiation is evolutionarily conserved across upper airway allergic inflammatory disorders, as supported by recent human upper airway immune endotyping data in patients with chronic rhinosinusitis with nasal polyps (CRSwNP)—a condition that shares overlapping type 2 inflammatory pathways and nasal epithelial dysfunction with AR (20). Nazari et al. demonstrated that in patients with the aspirin-exacerbated respiratory disease (AERD) subtype of CRSwNP, GATA3, IL-4 and IL-5 gene expression is markedly upregulated in nasal polyp tissues, with a pronounced Th2-skewed immune activation pattern linked to nasal epithelial dysfunction (20). This finding not only directly confirms that nasal epithelial cells act as central immune sentinels in driving the transcription and secretion of core Th2 cytokines, but also further underscores the universality of epithelial-immune crosstalk in mediating type 2 inflammation across upper airway allergic disorders. The sensing mechanism often involves the nuclear release of IL-33, which depends on apoptotic signaling pathways, highlighting a sophisticated regulatory system within epithelial cells to modulate immune activation based on the nature of the stimulus (21). Furthermore, epithelial PRRs can discriminate between different allergens and pathogens, enabling stimulus-specific immune responses that are tailored to the particular threat, rather than a generalized activation (22).

Upon recognition of allergens or pathogens, nasal epithelial cells secrete a complex milieu of cytokines, chemokines, and antimicrobial peptides that shape the local immune environment (23). The secretion of these molecules is tightly regulated by intracellular signaling networks downstream of PRR activation, including NF-κB and mitogen-activated protein kinase (MAPK) pathways, ensuring a balanced response that promotes pathogen clearance while limiting tissue damage (24, 25). Moreover, epithelial cells interact with resident immune sentinels such as macrophages and dendritic cells, which further amplify and tailor the immune response through antigen presentation and cytokine production (26). This crosstalk is crucial for bridging innate and adaptive immunity and for maintaining mucosal homeostasis. In AR, dysregulation of these processes leads to barrier dysfunction and chronic inflammation, underscoring the importance of epithelial immune sentinel functions in disease pathogenesis and potential therapeutic targeting (27–29).

Notably, the immune surveillance capacity of nasal mucosal epithelial cells is tightly coupled to intracellular Ca²^+^ signaling—this critical molecular link bridges allergen sensing and downstream pathological responses. When epithelial PRRs detect allergens or DAMPs, they trigger intracellular signaling cascades that converge on Ca²^+^-permeable ion channels. For example, TLR activation induces downstream G-protein-coupled receptor (GPCR) signaling, which in turn elevates intracellular Ca²^+^ concentrations via transient receptor potential vanilloid 1 (TRPV1) channel opening (30); at the same time, protease-containing allergens activate protease-activated receptors (PARs) on the nasal epithelial cell membrane, directly driving Ca²^+^ influx through store-operated Ca²^+^ entry (SOCE) channels (31, 32). This PRR-mediated Ca²^+^ signaling activation is not only a downstream outcome of immune surveillance by nasal epithelial sentinels, but also the primary trigger for subsequent epithelial barrier remodeling and inflammatory mediator secretion—this thus forms the initiating step of the Ca²^+^-mediated epithelial gatekeeper axis we propose in this review.

## 3 Conceptual advances beyond existing reviews

Here, we synthesize seminal work in the field, with a focused analysis of two influential reviews (7, 29) that have advanced the current understanding of nasal epithelial biology in the context of AR. These foundational reviews have collectively propelled the field forward by comprehensively characterizing the link between AR pathogenesis and nasal epithelial barrier dysfunction, including a detailed account of TJ molecule downregulation in both AR patients and preclinical models. They have also rigorously dissected multi-faceted regulatory factors governing this dysfunction, encompassing Th2 cytokines, epithelial-derived alarmins, epigenetic modifications, neuroimmune crosstalk, and environmental triggers. Notably, one review identified novel epithelial cell subsets and emphasized the “epithelial endotype” as a conserved feature across allergic airway diseases, while the other highlighted the genetic and epigenetic regulation of epithelial barrier integrity. Together, these two works have established a robust foundation for elucidating the contribution of nasal epithelial dysfunction to AR pathogenesis. Despite these advances, critical knowledge gaps persist: neither review identifies Ca²^+^ signaling as the central regulatory hub bridging epithelial “immune sentinel” function to TJ remodeling and inflammatory amplification. While transient receptor potential (TRP) channels were noted as potential mediators in this process, their specific role in driving Ca²^+^ influx and subsequent downstream signaling cascades was not defined. Furthermore, these works either conceptualized epithelial barrier impairment as a passive byproduct of inflammation or adopted a broad focus encompassing both upper and lower airways. This has resulted in an incomplete characterization of nasal epithelium-specific regulatory mechanisms and a lack of clarity regarding the molecular switch that initiates the “barrier damage–inflammation” vicious cycle, leaving key pathological processes fragmented rather than integrated into a cohesive mechanistic network. Building on these foundational contributions, the present review addresses these critical gaps by proposing the “Ca²^+^-mediated epithelial gatekeeper axis in AR”—a unifying framework that integrates allergen sensing, Ca²^+^ signaling activation, TJ remodeling, and inflammatory amplification into a continuous mechanistic cascade. We further define the dual regulatory role of Ca²^+^ signaling in preserving epithelial barrier integrity and driving inflammatory activation via the NF-κB/NLRP3 pathways, and link these molecular targets to clinical AR phenotypes through the integration of clinical and preclinical evidence, thereby strengthening the translational relevance of this mechanistic model.

## 4 Regulatory mechanisms of Ca^2+^ signaling pathway in nasal mucosal epithelial cells

As a key downstream signal of the nasal epithelial “immune sentinel” sensing pathway, Ca²^+^ signaling transduces allergen recognition into cellular functional responses, namely barrier regulation and inflammatory activation, through its unique spatiotemporal dynamics and the subsequent activation of downstream effector molecules. Activation of Ca²^+^ signaling downstream of PRRs, PAR-2 and PAR-4 depends on the coordinated activity of multiple Ca²^+^-permeable ion channels and regulatory molecules, whose basic biological properties and functional mechanisms we systematically elaborate in the subsequent sections.

### 4.1 Basic biological characteristics of Ca^2+^ signaling

Ca²^+^ channels (including voltage-gated Ca²^+^ channels, transient receptor potential (TRP) channels, stretch-activated channels (SAC), and SOCE channels), calcium pumps, and calcium-binding proteins are key components of Ca²^+^ signaling (33, 34). The versatility of Ca^2+^ signaling as a second messenger arises from its ability to generate spatially and temporally diverse Ca^2+^ signals or “calcium signatures” that can be localized within subcellular compartments or propagate as waves across cells (35, 36). Ca^2+^ signaling is further modulated by complex feedback mechanisms. For example, TRPM4 and TRPM5 channels convert intracellular Ca^2+^ increases into membrane depolarization, which in turn affects Ca^2+^ entry, illustrating a bidirectional interplay between electrical and chemical signals (37). Additionally, Ca^2+^ signaling is integrated with other signaling pathways such as cyclic nucleotides and AMPK, contributing to cellular metabolism and viral infection processes (38, 39). The compartmentalization of Ca^2+^ signals within organelles, such as mitochondria and peroxisomes, adds another layer of regulation influencing both organelle-specific and global cellular functions (40). These signals orchestrate a multitude of cellular processes through complex decoding mechanisms, underscoring calcium’s central role in cellular physiology and pathophysiology (41).

### 4.2 Relationship between Ca^2+^ signaling and epithelial barrier function

Ca^2+^serves as critical second messengers, modulating TJs assembly, disassembly, and remodeling through various signaling pathways (42). For instance, activation of G-protein coupled receptors (GPCRs) such as GPR120 can elevate intracellular Ca^2+^ levels, which subsequently triggers downstream effectors like myosin light chain kinase (43) (MLCK). MLCK phosphorylates myosin light chains, promoting actomyosin contraction that transiently increases paracellular permeability by modulating TJ protein distribution and function (44, 45). This mechanism was demonstrated in intestinal epithelial IPEC-J2 cells, where the c9, t11-conjugated linoleic acid (CLA) isomer activated GPR120, increased Ca^2+^, and stimulated MLCK signaling, resulting in decreased expression of TJ proteins and impaired barrier function both *in vitro* and *in vivo* (46). Ca^2+^ signaling is integral to the regulation of tight junction protein expression and function, thereby maintaining epithelial barrier integrity (47). Dysregulation of Ca^2+^ homeostasis, whether through environmental insults, inflammatory mediators, or pharmacological agents, can lead to TJ protein degradation, altered localization, and increased paracellular permeability (48, 49).

### 4.3 Ca^2+^ signal-mediated activation of inflammatory responses

Ca^2+^ signaling plays a pivotal role in the activation of key inflammatory pathways, including the NF-κB pathway and the NLRP3 inflammasome, thereby amplifying inflammation in allergic and other immune-mediated diseases (50, 51). However, this regulatory effect is not a simple linear causal relationship, but rather is governed by context-dependent cues, concentration/co-signaling thresholds, and intrinsic opposing regulatory circuits—key factors that collectively determine the activation status of the NF-κB/NLRP3 axis. First, context dependence is evident in microenvironmental and cell-type specificity: pro-inflammatory cytokines in the allergic nasal mucosal microenvironment can “sensitize” the pathway by potentiating the responsiveness of the NF-κB/NLRP3 axis to Ca²^+^ influx, while anti-inflammatory mediators such as IL-10 attenuate this responsiveness by downregulating STIM1 expression (52, 53). Cell-type specificity further shapes this regulatory process: in nasal mucosal epithelial cells, Ca²^+^ influx primarily drives NF-κB activation via the stromal interaction molecule 1 (STIM1)/calcium release-activated calcium modulator 1 (ORAI1) axis (30), whereas in immune cells such as macrophages, Ca²^+^-dependent NLRP3 activation requires synergistic TLR4 signaling to initiate this cascade (54, 55). Second, threshold effects are an essential requirement for pathway activation: intracellular Ca²^+^ concentrations in nasal epithelial cells must reach 100–300 nM to induce NF-κB nuclear translocation, and subthreshold Ca²^+^ fluctuations only sustain cellular homeostasis without eliciting inflammatory responses (30, 32); notably, house dust mites (HDM)-induced Ca²^+^ influx must reach a minimum of 150 nM to activate NF-κB (32). For NLRP3 inflammasome activation, Ca²^+^ influx alone is insufficient—ROS-mediated oxidative stress acts as a “second signal” to synergistically overcome this activation threshold, and the NLRP3 inflammasome remains inactive even at sufficient Ca²^+^ concentrations in the absence of ROS (50, 56). Third, multiple endogenous opposing regulatory pathways prevent excessive pathway activation: SARAF (Store-operated Ca²^+^ Entry-associated Regulatory Factor) interacts with STIM1 to limit SOCE-mediated Ca²^+^ influx, thus attenuating NF-κB activation (57); the Ca²^+^-dependent phosphatase PP2A dephosphorylates the p65 subunit of NF-κB, inhibiting its transcriptional activity and blocking pro-inflammatory cytokine secretion (58); in addition, IL-10 secreted following NLRP3 activation downregulates ORAI1 expression, reducing Ca²^+^ influx and forming a negative feedback loop (53). The intracellular Ca^2+^ increase acts as a second messenger that triggers a cascade of molecular events leading to pro-inflammatory gene expression and cytokine production (59). In immune cells, SOCE mediated stromal interaction molecule 1 (STIM1) and ORAI1 channels is essential for NF-κB signaling and inflammatory cytokine production (60). For example, lipopolysaccharide (LPS) stimulation upregulates STIM1/ORAI1 expression in bovine mammary epithelial cells, increasing Ca^2+^ influx and activating NF-κB, which promotes the release of pro-inflammatory mediators (54, 61). Inhibition of these Ca^2+^ channels attenuates NF-κB activation and reduces inflammation, highlighting the centrality of Ca^2+^ signaling in inflammatory amplification (52, 58).

## 5 Abnormal activation mechanism of Ca^2+^ Signaling in nasal mucosal epithelial cells in AR

### 5.1 Clinical evidence of abnormal Ca^2+^ signaling in nasal mucosal epithelial cells of AR patients

Clinical investigations have increasingly demonstrated that nasal mucosal epithelial cells from patients with AR exhibit significant abnormalities in Ca^2+^ signaling pathways, which are crucial in modulating epithelial barrier function and immune responses (30). Transcriptomic analyses of nasal epithelial cells from AR patients have revealed significantly elevated mRNA levels of these Ca^2+^ channel components compared to healthy controls, a finding further corroborated by protein expression studies, indicating a robust upregulation at both transcriptional and translational levels (62). This aberrant expression likely contributes to enhanced Ca^2+^ influx responses upon allergen exposure. Functional Ca^2+^ imaging studies provide direct evidence of altered Ca^2+^ signal dynamics in AR epithelial cells (55). This heightened Ca^2+^ sensitivity and altered kinetic profile suggest a hyperresponsive state of the epithelial Ca^2+^ signaling machinery in AR, which may potentiate downstream inflammatory cascades (63). The involvement of TRPV1 channels, which are upregulated in AR nasal mucosa and mediate Ca^2+^ influx leading to proinflammatory cytokine release such as IL-33, further supports the critical role of Ca^2+^ channels in epithelial immune activation (30). The dysregulated Ca^2+^ signaling not only disrupts epithelial barrier integrity but also amplifies the release of inflammatory mediators, creating a vicious cycle that exacerbates mucosal inflammation (64).

### 5.2 Allergen-induced aberrant activation of Ca^2+^ signaling

Allergen exposure, particularly to common triggers such as HDM and pollen, initiates aberrant Ca^2+^ signaling in nasal mucosal epithelial cells through receptor-mediated pathways, which plays a pivotal role in the pathogenesis of AR (63). Protease allergens from HDM, exhibit trypsin-like serine protease activity that activates protease-activated receptor-2 (PAR-2) on airway epithelial cells (31). This activation leads to an abnormal increase in intracellular Ca^2+^ levels, disrupting Ca^2+^ homeostasis and triggering downstream inflammatory cascades (65). Similarly, HDM allergens induce Ca^2+^ mobilization via PAR-2 and PAR-4, as well as TRPV1 channels, leading to increased intracellular cation levels and promoting alarmin release [IL-33 and Thymic Stromal Lymphopoietin (TSLP)] from epithelial cells in asthma patients (31). This aberrant Ca^2+^ signaling is closely associated with airway epithelial barrier dysfunction and inflammatory amplification (32). Beyond allergen-specific triggers, ambient air pollutants and irritants—including fine particulate matter (PM_2_._5_), volatile organic compounds (VOCs), and diesel exhaust particles (DEP)—also act as potent inducers of aberrant Ca²^+^ signaling in nasal epithelial cells (66). These chemical pollutants directly perturb Ca²^+^-permeable ion channels or modulate SOCE pathways in nasal epithelial cells, triggering unregulated Ca²^+^ influx that disrupts intracellular Ca²^+^ homeostasis (67, 68). This dysregulation subsequently activates pro-inflammatory signaling cascades and impairs tight junction integrity, creating a pro-inflammatory microenvironment that favors the initiation and progression of allergic responses (69). Notably, these environmental stimuli often act synergistically with allergens: they can “prime” nasal epithelial cells to enhance their responsiveness to subsequent allergen exposure, leading to exaggerated Ca²^+^ influx and alarmin release, thereby exacerbating AR severity (70, 71).

In addition to chemical pollutants, physical environmental factors such as ambient temperature and humidity also modulate Ca²^+^ signaling dynamics (72). Extreme temperature changes directly activate temperature-sensitive Ca²^+^ channels in nasal epithelial cells, inducing Ca²^+^ influx that promotes the release of pro-inflammatory mediators and neuropeptides, which in turn disrupt epithelial barrier function and enhance inflammatory cell recruitment (73). Conversely, abnormal ambient humidity alters epithelial hydration status, leading to intracellular Ca²^+^ overload that triggers cellular stress responses or apoptotic pathways in nasal epithelial cells (74). These findings highlight that environmental factors are not merely passive co-factors but active modulators of the Ca²^+^-mediated epithelial gatekeeper axis, capable of both initiating and amplifying the pathological cascade in AR.

## 6 The effect of Ca^2+^ signal regulation imbalance on the progression of AR disease

### 6.1 Aberrant Ca^2+^ signaling directly impairs nasal mucosal epithelial barrier integrity

The integrity of the nasal mucosal epithelial barrier is critically dependent on the precise regulation of Ca^2+^ signaling, which orchestrates tight junction assembly and epithelial cell survival (75). Disruption of Ca^2+^ homeostasis, particularly abnormal Ca^2+^ influx, initiates a cascade of molecular events that compromise barrier function (76, 77). One key mechanism involves the activation of the Ca^2+^-dependent phosphatase calcineurin (CaN), which aberrantly modifies tight junction proteins such as occludin and zonula occludens-1 (ZO-1) (78). Elevated intracellular Ca^2+^ levels lead to hyperphosphorylation of these proteins, causing their dissociation from the cell membrane and subsequent degradation (79). This process results in the widening of intercellular spaces and loss of epithelial barrier continuity (80, 81). Concurrently, sustained high intracellular Ca^2+^ triggers mitochondrial apoptotic pathways, promoting caspase-3 activation and accelerating epithelial cell apoptosis (82, 83). The increased epithelial cell shedding further exacerbates barrier disruption, facilitating allergen penetration and inflammatory cell infiltration (84, 85). These findings underscore the direct deleterious impact of Ca^2+^ signaling abnormalities on nasal epithelial barrier function, highlighting a pivotal role of Ca^2+^ dysregulation in the pathogenesis of barrier impairment in AR (86).

### 6.2 Inflammatory cytokines feedback to exacerbate Ca^2+^ signal abnormalities: formation and amplification of a vicious cycle

**Figure 1:**
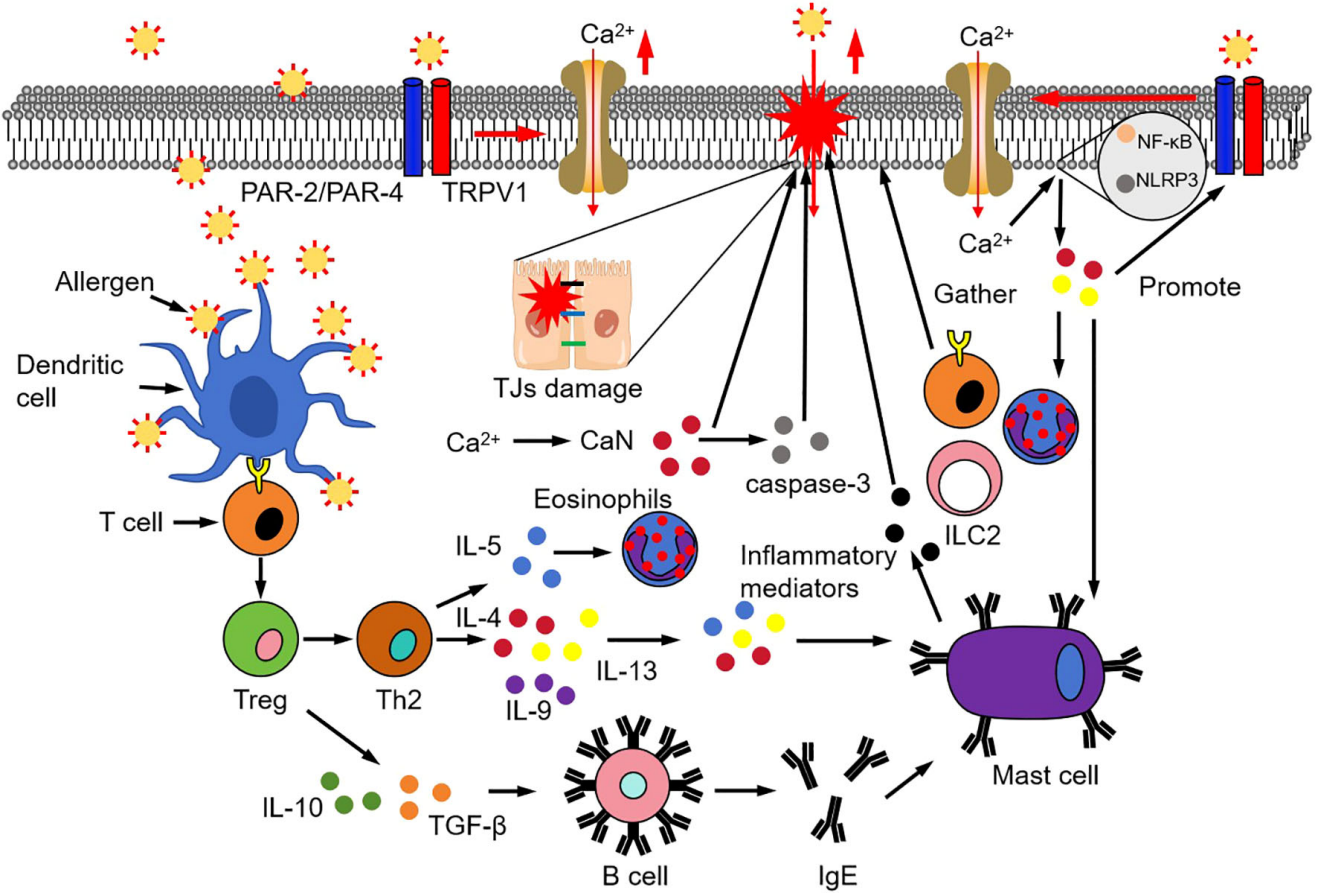
Schematic diagram of Ca²^+^ signaling-mediated barrier damage and inflammatory amplification loop in allergic rhinitis (AR). This figure illustrates two linked pathological processes: (1) Allergens bind to protease-activated receptor 2/4 (PAR-2/PAR-4) and transient receptor potential vanilloid 1 (TRPV1) on nasal epithelial cells, triggering Ca²^+^ influx via stromal interaction molecule 1 (STIM1)/calcium release-activated calcium modulator 1 (ORAI1) channels. Elevated intracellular Ca²^+^ activates calcineurin (inducing tight junction [TJ] protein degradation) and caspase-3 (promoting epithelial apoptosis), disrupting the barrier and enabling allergen penetration. (2) Aberrant Ca²^+^ signaling activates nuclear factor κB (NF-κB)/NLR family pyrin domain containing 3 (NLRP3) pathways, driving release of IL-33 and T helper type 2 (Th2) cytokines (IL-4, IL-5, IL-13). These mediators recruit eosinophils and group 2 innate lymphoid cells (ILC2s), whose secreted factors feedback to upregulate epithelial Ca²^+^ channels, forming a self-sustaining vicious cycle that perpetuates inflammation and AR symptoms.

Ca^2+^ signaling not only mediates epithelial barrier integrity but also profoundly influences the expression and release of key Th2-type cytokines, such as IL-4, IL-5, and IL-13, which are central to allergic inflammation (87, 88). Elevated intracellular Ca^2+^ levels activate transcriptional pathways that upregulate these cytokines, thereby promoting the type 2 inflammatory milieu characteristic of AR. Moreover, Ca^2+^-dependent mechanisms regulate the secretion of chemokines like CCL11 (eotaxin-1) and CCL26 (eotaxin-3), which are potent chemoattractants for eosinophils and other inflammatory cells, facilitating their recruitment to the nasal mucosa (89). Importantly, the sustained Ca^2+^ signal abnormalities maintain continuous release of inflammatory mediators, establishing a self-amplifying loop that reinforces barrier dysfunction and inflammation (90). This vicious cycle is further supported by evidence that targeting Ca^2+^ signaling pathways can attenuate inflammatory cytokine production and cellular activation, suggesting potential therapeutic avenues (56, 91). In chronic inflammatory states, dysregulated Ca^2+^ signaling emerges as a critical node that sustains and amplifies the inflammatory microenvironment, making Ca^2+^ channels, pumps, and their regulators promising targets for intervention to disrupt this deleterious feedback loop (92) (**Figure 1**).

## 7 Therapeutic potential of AR targeting Ca^2+^ signaling regulation in nasal mucosal epithelial function

### 7.1 Intervention effects and mechanisms of Ca^2+^ channel blockers

Calcium Release-Activated Calcium (CRAC) channel inhibitors: Selective inhibitors such as GSK-7975A and YM-58483 target the STIM1/Orai1 interaction, and have shown promising preclinical efficacy in AR mouse models by reducing epithelial permeability and suppressing IL-33 secretion (93). However, their clinical translation potential remains limited to date: first, no Phase II/III clinical trials have been performed in AR patients to assess these agents, and most efficacy data come from animal models and *in vitro* nasal epithelial cell assays (93, 94); second, significant safety concerns remain—non-selective CRAC channel inhibition may disrupt physiological Ca²^+^ signaling in non-target tissues, with the potential to induce adverse events like cardiac arrhythmias and neurotoxicity (95). While STIM1/Orai1-selective inhibitors are developed to reduce these off-target effects (95), their long-term safety profiles are largely undefined. Specificity is thus a key consideration in the design of CRAC channel inhibitors, as different Orai isoforms have distinct regulatory properties: Orai1 and Orai2 are sensitive to intracellular pH fluctuations and dependent on STIM1, while Orai3 is not subject to this STIM1-mediated regulation. This means selective targeting of the STIM1/Orai1 interaction can yield therapeutic effects without globally disrupting physiological Ca²^+^ signaling (95). In addition, agents like YM-58483 have been shown to boost cell viability and increase junctional protein expression in nasal epithelial cells under inflammatory conditions, a finding that further supports their preclinical potential to preserve epithelial barrier integrity (94). Apart from direct small-molecule inhibition of CRAC channels, SPLUNC1-derived peptides can induce Orai1 internalization and subsequent lysosomal degradation, thus effectively reducing SOCE and suppressing inflammatory responses *in vitro* (96). This points to a promising alternative strategy for targeting STIM1/Orai1 function, though the *in vivo* stability and bioavailability of these peptide agents need further optimization (57).

Transient Receptor Potential Canonical (TRPC) channel inhibitor SKF-96365 corrects aberrant Ca²^+^ signaling in nasal mucosal epithelial cells from AR patients by inhibiting TRPC6 channel activity, and exhibits symptom-relieving effects in murine AR models (97, 98). However, its clinical translatability is limited by key drawbacks: first, SKF-96365 is a non-selective TRPC inhibitor that also targets TRPC3/4/5 channels alongside TRPC6 (99), and this lack of selectivity can disrupt physiological Ca²^+^-dependent processes in nasal epithelial cells, potentially causing adverse effects such as nasal dryness and impaired pathogen clearance at the nasal mucosa (100); second, clinical evidence for AR is very limited, with all current data restricted to *in vitro* cell assays and small animal models, and no large-scale human trials have been performed to confirm its efficacy and safety in AR patients (98, 101). Dysregulation of TRPC6-mediated Ca²^+^ signaling is a major contributor to pathological processes linked to abnormal cell proliferation, excessive inflammation, and epithelial barrier dysfunction (100, 102). While SKF-96365 acts as a potent pharmacological tool that modulates TRPC6 channel activity and restores Ca²^+^ signaling homeostasis in preclinical models, its lack of selectivity and corresponding clinical translational potential for AR are yet to be validated (99, 101).

### 7.2 Targeted blocking strategies of the inflammatory factor - Ca^2+^ signaling axis

Neutralizing IL-33 using specific monoclonal antibodies reverses excessive Ca²^+^ influx and subsequent signaling dysregulation in nasal epithelial cells, thereby restoring the expression and function of tight junction proteins in preclinical models (30, 103). Occludin is critical for maintaining epithelial tight junction integrity, and its reduced expression correlates closely with increased epithelial permeability and greater allergen penetration across the mucosal barrier. Restoring occludin expression after IL-33 blockade reinforces the nasal epithelial barrier, reducing allergen-induced immune activation and breaking the vicious cycle of inflammatory amplification (104, 105). However, IL-33 targeting with monoclonal antibodies has key clinical limitations for AR treatment: first, IL-33 inhibition is highly effective only in Th2-predominant AR subtypes, and has little to no efficacy in non-Th2 AR phenotypes (103); second, safety risks include a higher risk of upper respiratory tract infections due to impaired mucosal innate immunity (103), and the long-term risks of immune dysregulation in AR patients remain unassessed; third, tozorakimab has demonstrated encouraging efficacy in Phase II trials for CRSwNP (103), but no dedicated Phase III trials have been done to confirm its efficacy in AR patients, and extrapolating efficacy across these diseases is not a reliable approach. These findings confirm that excessive IL-33-driven Ca²^+^ signaling activation contributes to nasal epithelial barrier dysfunction in Th2-skewed AR (103). Furthermore, bispecific anti-IL-4/IL-13 antibodies exert their therapeutic effects in part by reducing Ca²^+^ channel expression on nasal epithelial cells; this process is critical for regulating intracellular Ca²^+^ signaling that controls nasal epithelial barrier integrity and the secretion of inflammatory mediators (106). By lowering Ca²^+^ channel expression, these antibodies reduce Ca²^+^-dependent activation of nasal epithelial cells, which in turn decreases the secretion of proinflammatory mediators such as thymus and activation-regulated chemokine (TARC) and other Th2-associated cytokines. That said, their clinical use in AR is limited by several key issues: high treatment costs that could limit clinical access; suboptimal response rates in patients with mild-to-moderate AR (106); and potential adverse events like injection-site reactions and eosinophilia, which call for long-term clinical monitoring (106).

### 7.3 Therapeutic challenges and future perspectives

Despite compelling preclinical evidence supporting Ca²^+^ signaling as a novel therapeutic target for AR, several key barriers hinder its clinical translation. First, a lack of AR-specific targeted agents: most available Ca²^+^ channel inhibitors and antibodies targeting inflammatory factors are not selective for nasal epithelial cells or distinct AR endotypes, leading to off-target effects and limited efficacy in the heterogeneous AR patient population. Second, insufficient clinical evidence: most efficacy data comes from *in vitro* experiments and small-animal models, and there have been few large-scale, long-term clinical trials in AR patients to confirm safety, optimal dosing, and durability of response. Third, achieving a favorable safety-efficacy balance remains a challenge: although targeting Ca²^+^ signaling or related inflammatory mediators can alleviate AR-related inflammation, this can disrupt physiological Ca²^+^-dependent processes in the nasal mucosa, which increases the risk of mucosal infections or epithelial dysfunction. Fourth, a lack of biomarker-guided precision therapy: currently, there are no validated predictive biomarkers to identify and stratify AR patients who are most likely to benefit from Ca²^+^-targeted therapies, leading to the potential for overtreatment in non-responsive patients.

Importantly, the impact of environmental factors on Ca²^+^ signaling also has critical implications for therapeutic development. The modulatory effects of ambient pollutants and physical factors on Ca²^+^ signaling dynamics can significantly alter the responsiveness of nasal epithelial cells to Ca²^+^-targeted interventions, thereby influencing both therapeutic efficacy and safety profiles. This context-dependent variability necessitates a more personalized approach to treatment, where therapeutic strategies are tailored to account for individual environmental exposure histories. Future studies should therefore incorporate environmental exposure data into clinical trial design, to better stratify patients and optimize therapeutic outcomes. Future research should thus prioritize the following key areas: (1) developing nasal mucosa-targeted Ca²^+^ channel modulators to minimize systemic adverse effects; (2) designing and conducting endotype-specific clinical trials to confirm the efficacy of these targeted agents; (3) identifying and validating predictive biomarkers to enable rational patient stratification for Ca²^+^-targeted therapy; (4) exploring rational combination therapies to achieve synergistic therapeutic effects, while lowering individual drug doses and their corresponding adverse effects. Overcoming these key barriers will allow Ca²^+^ signaling-targeted strategies to reach their full therapeutic potential as novel treatment options for AR.

## 8 Discussion

The role of nasal mucosal epithelial cells as “immune sentinels” in AR represents a critical nexus in understanding the disease’s pathogenesis and progression (107, 108). From an expert perspective, this review underscores the central importance of Ca^2+^ signaling pathways in regulating both the epithelial barrier function and local immune responses. The intricate balance maintained by Ca^2+^ signals ensures mucosal integrity and appropriate immune activation; however, disruptions in this signaling cascade precipitate epithelial barrier dysfunction and amplify inflammatory circuits, thereby perpetuating a chronic inflammatory milieu that exacerbates disease severity.Nasal mucosal epithelial cells act as critical “immune sentinels” in AR, with their function representing a key nexus for understanding the disease’s pathogenesis and progression. In summary, this review highlights the central role of Ca²^+^ signaling pathways in coordinating both nasal epithelial barrier function and local nasal mucosal immune responses. The delicate spatiotemporal balance of Ca²^+^ signaling maintains nasal mucosal integrity and coordinates appropriate immune activation; in contrast, dysregulation of this signaling cascade leads to epithelial barrier dysfunction and amplifies inflammatory pathways, in turn maintaining a chronic inflammatory microenvironment that exacerbates AR severity.

The development of research in this area has significantly advanced our comprehension of AR beyond a simplistic view of immune hypersensitivity. It highlights a nuanced interplay between epithelial cells and immune effectors mediated through Ca^2+^-dependent mechanisms. This evolving paradigm bridges molecular insights with clinical manifestations, offering a more integrated understanding of disease dynamics. Nevertheless, the complexity of Ca^2+^ signaling—encompassing diverse channels, pumps, and downstream effectors—poses challenges in reconciling disparate findings across studies.Notably, Ca²^+^ influx does not regulate the NF-κB/NLRP3 axis through a unidirectional causal relationship; instead, this regulation depends on the inflammatory microenvironment and cell-type specificity, requires sufficient intracellular Ca²^+^ levels and synergistic signals to hit activation thresholds, and is further modulated by intrinsic negative feedback mechanisms such as SARAF, PP2A, and IL-10. Some research emphasizes specific Ca^2+^ channels’ roles, while others focus on broader signaling networks or cross-talk with other intracellular pathways. Balancing these perspectives requires a holistic approach that appreciates both the specificity of molecular targets and the systemic context in which they operate.

Critically, aberrant Ca^2+^ signaling not only compromises the epithelial barrier but also fuels a self-perpetuating inflammatory loop, underscoring its dual role in both initiating and sustaining allergic inflammation (53, 55). This duality presents both challenges and opportunities for therapeutic intervention. Targeting Ca^2+^ signaling pathways offers a promising avenue for novel treatments aimed at restoring barrier integrity and modulating immune responses simultaneously. Such strategies could potentially interrupt the vicious cycle of inflammation characteristic of AR, thereby improving patient outcomes.

Future research must prioritize the precise modulation of Ca^2+^-related molecular targets to translate these mechanistic insights into effective clinical therapies. This entails rigorous characterization of Ca^2+^ signaling components in nasal epithelial cells under both physiological and pathological conditions, as well as the development of selective modulators with minimal off-target effects. Additionally, integrating emerging technologies such as single-cell transcriptomics and advanced imaging can deepen our understanding of the spatiotemporal dynamics of Ca^2+^ signaling in the nasal mucosa.

Ultimately, advancing targeted Ca^2+^ signaling interventions holds the promise of not only mitigating AR symptoms but also enhancing patients’ quality of life by addressing the disease’s underlying pathophysiology. As the field progresses, a multidisciplinary approach combining molecular biology, immunology, pharmacology, and clinical research will be essential to fully harness the therapeutic potential of Ca^2+^ signaling modulation. This balanced and comprehensive perspective will drive the evolution of personalized medicine strategies tailored to the complex immunological landscape of AR.
